# EEG sharp waves are a biomarker of striatal neuronal survival after hypoxia-ischemia in preterm fetal sheep

**DOI:** 10.1038/s41598-018-34654-7

**Published:** 2018-11-05

**Authors:** Hamid Abbasi, Paul P. Drury, Christopher A. Lear, Alistair J. Gunn, Joanne O. Davidson, Laura Bennet, Charles P. Unsworth

**Affiliations:** 1 Department of Engineering Science, The University of Auckland, Auckland, New Zealand; 2 Department of Physiology, Faculty of Medical and Health Sciences, The University of Auckland, Auckland, New Zealand

## Abstract

The timing of hypoxia-ischemia (HI) in preterm infants is often uncertain and there are few biomarkers to determine whether infants are in a treatable stage of injury. We evaluated whether epileptiform sharp waves recorded from the parietal cortex could provide early prediction of neuronal loss after HI. Preterm fetal sheep (0.7 gestation) underwent acute HI induced by complete umbilical cord occlusion for 25 minutes (n = 6) or sham occlusion (control, n = 6). Neuronal survival was assessed 7 days after HI by immunohistochemistry. Sharp waves were quantified manually and using a wavelet-type-2-fuzzy-logic-system during the first 4 hours of recovery. HI resulted in significant subcortical neuronal loss. Sharp waves counted by the automated classifier in the first 30 minutes after HI were associated with greater neuronal survival in the caudate nucleus (r = 0.80), whereas sharp waves between 2–4 hours after HI were associated with reduced neuronal survival (r = −0.83). Manual and automated counts were closely correlated. This study suggests that automated quantification of sharp waves may be useful for early assessment of HI injury in preterm infants. However, the pattern of evolution of sharp waves after HI was markedly affected by the severity of neuronal loss, and therefore early, continuous monitoring is essential.

## Introduction

Worldwide, hypoxic-ischemic (HI) intrapartum insults were associated with approximately 1,150,000 cases of HI encephalopathy in 2010, 8.5 per 1,000 live births, and in turn with high rates of death and neurodevelopmental disability^1^. Rates of HI are particularly high after premature birth^2^, and there are no established neuroprotective therapies. It is now well established that postnatal neuroprotective treatment is potentially viable after perinatal HI, because brain injury evolves over time^3,4^. Clinical and pre-clinical studies of HI in preterm and term fetuses and newborns, show that after reperfusion there is a latent phase of recovery of oxidative metabolism lasting up to 6–15 hours, followed by a phase of secondary deterioration over several days where oxidative metabolism fails and the majority of cell death occurs^5,6^.

Therapeutic hypothermia after HI is neuroprotective in both preterm and term animals^7,8^, and is now standard care for term newborns after moderate to severe HI^9^. Hypothermia, and potentially other treatments that act through similar pathways, is only effective when started during the latent phase after HI; efficacy is rapidly lost with increasing delay after HI as previously reviewed^3^. Clinically, the precise timing of HI before birth is often difficult to determine particularly in preterm infants^10^, and so by the time that any treatment can be started the injury may have evolved beyond the window of opportunity for treatment^11^.

Biological markers (biomarkers) are essential to help identify infants who are at risk of injury, and critically, whether affected infants are still in a stage of evolving neural injury when they would benefit from early neuroprotective interventions^12,13^. Magnetic resonance spectroscopy can determine changes in cerebral oxidative state, but cannot be used continuously, requires sick infants to be transported and is not available in many hospitals^14^. Similarly, plasma or urine biomarkers require intermittent sampling and have not yet been shown to discriminate phases of injury^12^.

Electroencephalographic (EEG) and amplitude integrated EEG (aEEG) monitoring within 6 hours after HI can predict neurological outcome in term infants, but in many mild and moderate cases may only be a reliable indicator towards the end of the latent phase, when the efficacy of early therapies such as therapeutic hypothermia is becoming limited^13,15–17^. There is encouraging evidence from meta-analysis that EEG and aEEG have potential predictive value in preterm infants. However, the evidence is heterogeneous and there have been few studies of early recordings^18^. Detailed examination of continuous EEG recordings can potentially provide additional information. For example, we have shown in preterm fetal sheep that during the latent phase there is intense epileptiform transient activity, including spikes, sharps, slow-waves, which correlated with subcortical neuronal loss^6,19–25^. There is clinical evidence that in the neonatal period these waveforms are associated with increased risk of disability^26,27^.

Clinical use of this finding will require automated quantification of numbers of transients, in contrast with visual assessment and counting in early studies^6,28,29^. Further, the predictive value in different epochs during the latent phase is unclear. In the present study, we sought to determine the earliest time at which sharp waves would predict adverse neurological outcomes after HI induced by reversible umbilical cord occlusion in preterm fetal sheep at 0.7 gestation, when brain maturation is broadly equivalent to humans at 28–30 weeks of gestation^30^. We quantified numbers of sharp waves in defined epochs of time during the first 4 hours after HI by combining a wavelet-type-2-fuzzy logic system (WT-Type-2-FLS) for automatic identification of sharp waves^24^, and a stereotypic evolving micro-scale seizure detection method which helps to reduce false detection of sharp waves^23^. Numbers of sharp waves within each epoch were correlated with neuronal survival assessed 7 days after HI *in utero*.

## Materials and Methods

### Ethics

All animal procedures were approved by the Animal Ethics Committee of the University of Auckland and were in accordance with the Animal Welfare Act (1999) of New Zealand.

### Subjects

Twelve singleton Romney/Suffolk cross fetal sheep were surgically instrumented at 98–100 days of gestation (term is ~147 days) as previously described^31^. Food, but not water was withdrawn 18 hours before surgery. Ewes were given long acting oxytetracycline (20 mg/kg, Phoenix Pharm Distributors Ltd., Auckland, New Zealand) intramuscularly 30 minutes before surgery for prophylaxis. Anesthesia was induced by i.v. injection of propofol (5 mg/kg, AstraZeneca plc, London, UK) and general anesthesia was maintained using 2–3% isoflurane in oxygen. The depth of anesthesia, maternal heart rate and respiration were constantly monitored by trained anesthetic staff. Ewes received a constant infusion of isotonic saline (approximately 250 ml/hour) to maintain fluid balance.

### Surgical instrumentation

A midline abdominal incision was made to expose the uterus, and the fetus was partially exteriorized for instrumentation. Polyvinyl catheters (SteriHealth, Dandenong South, Victoria, Australia) were placed in a fetal femoral and brachial artery to measure blood pressure and for pre-ductal blood sampling. An additional catheter was placed into the amniotic sac for measurement of amniotic fluid pressure. Electrodes (AS633-5SSF, Cooner Wire, Chatsworth, CA, USA) were placed subcutaneously across the chest for measurement of the fetal electrocardiogram to derive fetal heart rate (FHR). Two pairs of electrodes (Cooner Wire) were placed on the dura over the parasagittal parietal cortex bilaterally, 10 and 15 mm anterior, 5 mm lateral to bregma to measure fetal EEG activity. An inflatable silicone occluder (*In Vivo* Metric, Healdsburg, California, United States) was placed around the umbilical cord to facilitate umbilical cord occlusion. Before closing the uterus, the antibiotic gentamicin (80 mg, Pfizer, Auckland, New Zealand) was administered into the amniotic sac along with 250 ml of sterile saline to replace lost amniotic fluid. The maternal midline skin incision was infiltrated with a local analgesic, 10 ml of 0.5% bupivacaine plus adrenaline (AstraZeneca) to provide long-acting analgesia. All fetal leads were exteriorized through the maternal flank and a maternal long saphenous vein was catheterized for post-operative care and euthanasia.

### Post-surgical care

After surgery ewes were housed in separate metabolic cages in a temperature-controlled room (16 ± 1 °C, humidity 50 ± 10%) with a 12 hour light/dark cycle (6.30 am lights on) and *ad libitum* access to water and food. Ewes and fetuses were allowed to recover for 4 days before experiments commenced. Ewes received i.v. antibiotics daily for four days (600 mg benzylpenicillin sodium; Novartis Ltd, Auckland, N.Z., and 80 mg gentamicin sulfate). Catheters were maintained patent by continuous infusion of heparinized saline (20 U/ml at 0.2 ml/hour).

### Experimental protocol

Experiments began between 9:00 and 9:30 am. Fetuses were randomly assigned to either the control (n = 6) or asphyxia (n = 6) groups. Fetal asphyxia was induced by complete umbilical cord occlusion for 25 minutes^31,32^. Control fetuses received no occlusion. Successful occlusion was verified through FHR and mean arterial blood pressure (MAP) recordings, corrected for maternal movement by subtraction of amniotic fluid pressure, and blood samples for pH, blood gases (ABL 800, Radiometer, Copenhagen, Denmark) and glucose and lactate levels (YSI model 2300, Yellow Springs, OH, USA). Fetal pre-ductal arterial blood samples were taken 15 minutes before, 5 and 17 minutes during, and 10 minutes, 1, 2, 4 and 6 hours after asphyxia and then once daily thereafter. Physiological parameters were recorded continuously from 24 hours before until the end of experiment 7 days after asphyxia when ewes and their fetuses were killed by an i.v. overdose of sodium pentobarbitone (9 g; Pentobarb 300, Chemstock International, Christchurch, NZ) and the fetal brains processed for histological analysis.

### Data acquisition and EEG analysis

All signals were processed using customized Labview-based data acquisition software (National Instruments, Austin, TX, USA). The analogue EEG signals were amplified 10,000× and processed with a sixth order low-pass Butterworth anti-aliasing filter with cut-off frequencies at 1.6 Hz and 512 Hz. The raw EEG signal was processed through an inverse Chebyshev low-pass filter with a cut-off frequency of 512 Hz, and saved at 1024 Hz for analysis of the raw EEG waveforms^24^. Digitized data were then extracted using MATLAB ((R2015b pro–64 bit, version 8.6.0.267246, Mathworks Inc., MA, USA). The data sets were initially zero-meaned and a 100^th^ order digital band-pass finite impulse response (FIR) filter with a normalized stop-band frequency (ω) between 0.05 and 0.13 was used to remove any remaining noise from the data.

**Figure 1:**
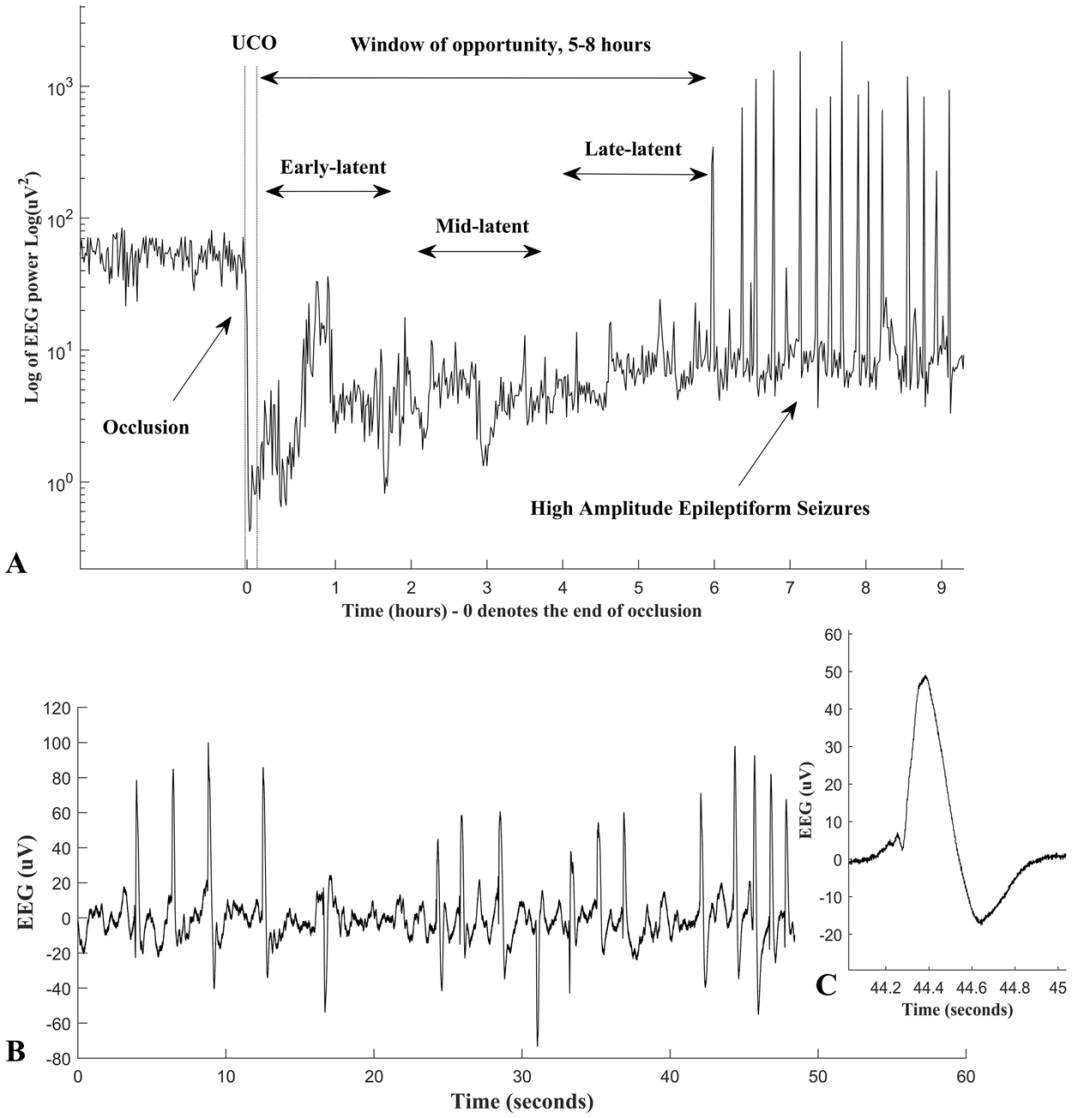
Panel A: shows an example of a raw EEG trace from one fetus taken from 3 hours before until 9 hours after an asphyxial insult (dotted lines) induced by complete umbilical cord occlusion (UCO). The 2 hour early, mid and late epochs of the latent phase are shown. Panel B: shows an example of repetitive single sharp waves in the raw EEG, data are from the mid-latent phase. Panel C: an example of a sharp wave after HI, showing the duration and characteristic shape of these wave forms.

We excluded the final 2 hours of the 6 hour recording in this study (the “late-latent” phase), due to the emergence of high amplitude stereotypic evolving seizures in many animals, similarly to our recent report.^33^ These were spectrally similar to the sharp waves of interest and so obstructed the assessment of the data in this sub-phase for several cases. Thus, data were analyzed in two epochs: the early-latent phase (0–2 h) and the mid-latent phase (2–4 h) (Fig. 1). In this study, we quantified sharp waves, occurring both singly and in repetitive series of multiple waves. Sharp waves were defined as epileptiform events that were of 70 to 250 ms duration, greater than 20 µV, and occurring on a suppressed EEG background (as illustrated in Fig. 1). Numbers of sharp waves were determined in temporal epochs of 2 hours, 1 hour, and 30 and 10 minutes. Empirically, 10 minutes was the minimum epoch in which significant correlations could be demonstrated.

Numbers of sharp waves in each epoch were determined both using an algorithm as described next (“automated counts”), and manually by an expert (HA) (“manual counts”).

### Combined WT-Type-2-Fuzzy classifier and stereotypic evolving micro-scale seizure detection

Numbers of sharp waves were quantified by a computer algorithm that combined a WT-Type-2-Fuzzy classifier, to detect sharp waves, both singly and in trains, with our stereotypic evolving micro-scale seizure filter to reduce false-positive detection of sharp waves, as previously demonstrated^23^. The stereotypic evolving micro-scale seizures observed in the latent phase after HI are continuous ongoing oscillations with frequency of 1.8–3 Hz (delta range), amplitude >10 µV. The duration of individual waves shows symmetrical sinusoidal distribution about the average of the signal, and continue for variable lengths; from less than 5–10 seconds to longer intervals of greater than 10 seconds and sometimes even longer intervals up to 70–90 seconds (for more information on stereotypic evolving micro-scale seizures, see^23^). These characteristics can be used to distinguish micro-scale seizures from sharp waves. For more information please see^23^ and^24^.

We used a Gaussian-2 mother wavelet at scale 32 wavelet transform of the raw sharp waves, sampled at 1024 Hz, to provide a robust ‘Footprint of Uncertainty’ and build compacted ‘Membership Functions’ that served to significantly improve the performance of the Type-2 Fuzzy logic classifiers for detection of sharp waves (with an overall performance of 97.4 ± 0.9%)^24^. Sensitivity and selectivity measures were calculated the performance of the classifiers.

### Immunohistochemistry

**Figure 2:**
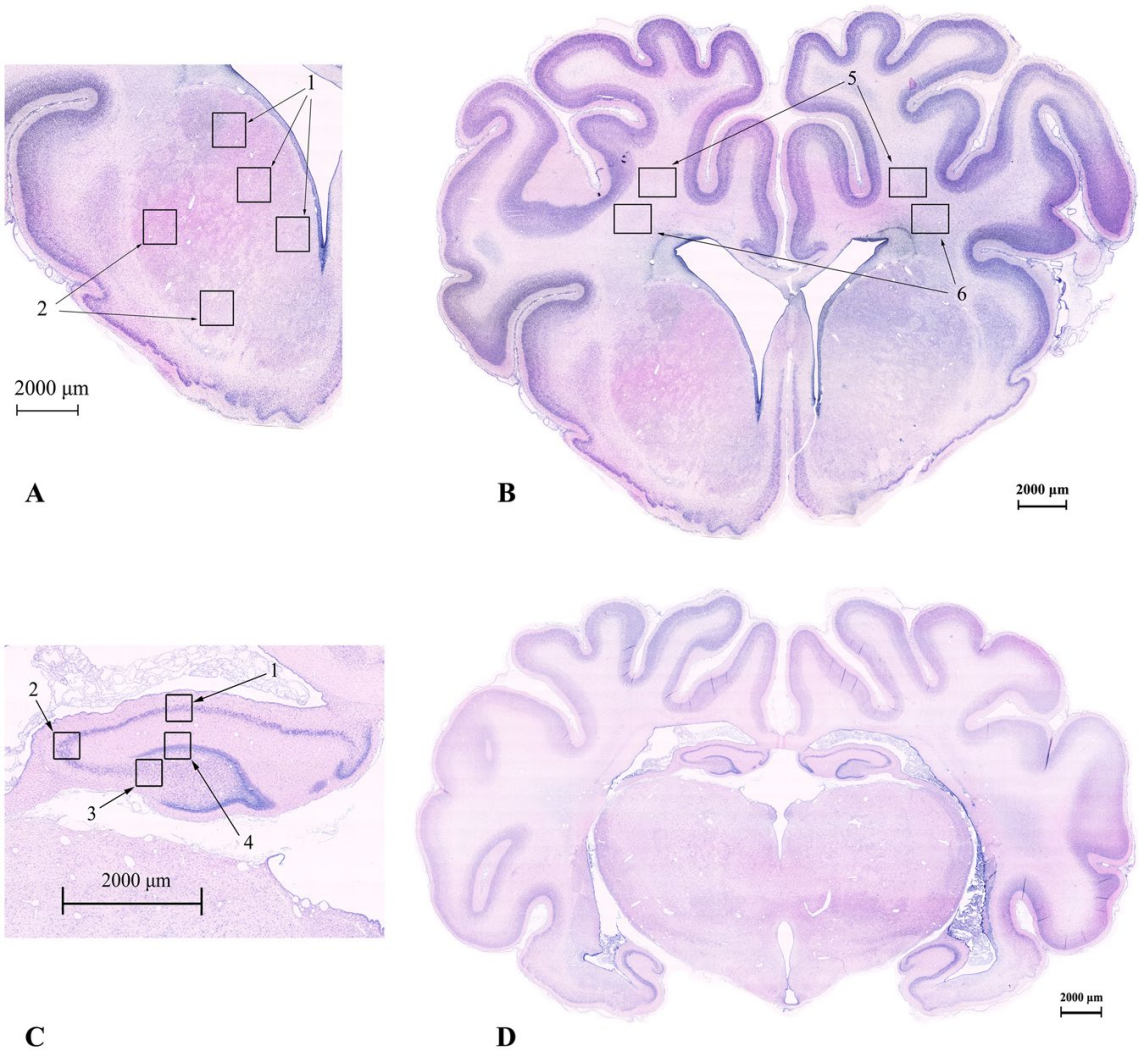
Photomicrographs of a preterm fetal sheep brain showing acid-fuchsin/thionin stained examples of the coronal brain sections used for histological analysis. (**A**) Areas sampled in the striatum including the caudate (1) and putamen (2) and in the two white matter tracts: the intragyral white matter (5) and periventricular white matter (6). A full section showing the striatum is shown in (**B**). (**C**) Areas sampled in the hippocampus including the CA1-2 (1), CA3 (2), CA4 (3) and DG (4) regions. A full section showing the hippocampus is shown in (**D**). Scale = 2000 μm.

Fetal brains were perfusion fixed *in situ*, flushed with 500 ml of 0.9% saline followed by 1 L of 10% phosphate buffered formalin. Brains were immersion fixed for a further 5–7 days, and then embedded in paraffin. Coronal slices (10 μm thick) were cut using a microtome (Leica Jung RM2035, Solms, Hessen, Germany) starting at the level of the dorsal hippocampus. Sections were stained with acid-fuchsin/thionin to assess gross morphological changes by light microscopy^34^; representative sections are shown Fig. 2 to illustrate the regions used for subsequent cell counting.

On separate sections, antigen retrieval was performed using the pressure cooker method with citrate buffer. Endogenous peroxidase quenching was performed by incubation in 1% H202 in methanol for anti-neuronal nuclei monoclonal antibody (NeuN, a marker of most neurons in the central nervous system^35^), ionized calcium binding adaptor molecule 1 (Iba1, a microglia/macrophage-specific calcium-binding protein) and 2′,3′-Cyclic-nucleotide 3′-phosphodiesterase (CNPase, a marker for mature and immature oligodendrocytes), and PBS for Olig-2 (a marker for oligodendrocytes at all stages of the lineage^36^). Blocking was performed in 3% normal horse serum (NHS) for NeuN and Iba1 and normal goat serum (NGS) for Olig-2, and CNPase for 1 hour at room temperature. Sections were labelled with 1:400 mouse NeuN (Chemicon International, Temecula, CA, USA), 1:400 Olig-2 (Chemicon International), 1:200 goat anti-Iba1 (Abcam) and 1:200 mouse anti-CNPase (Abcam) overnight at 4 °C. Sections were incubated in biotin-conjugated secondary 1:200 horse anti-mouse (NeuN) or 1:200 goat anti-rabbit IgG (Vector Laboratories, Burlingame, USA) in 3.5% NHS of NGS for goat. Slides were then incubated in ExtrAvidin® (1:200, Sigma-Aldrich Pty. Ltd.) in PBS for two hours at room temperature and then reacted in diaminobenzidine tetrachloride (DAB) (Sigma-Aldrich Pty. Ltd.). The reaction was stopped by washing in PBS, the sections dehydrated and mounted.

Brain regions of the forebrain used for analysis included the mid-striatum (comprising the caudate nucleus and putamen), and the frontal subcortical white matter (comprising the intragyral, IGWM, and periventricular, PVWM, regions) on sections taken 23 mm anterior to stereotaxic zero (Fig. 2). The cornu ammonis (CA) of the dorsal horn of the anterior hippocampus (divided into CA1/2, CA3, CA4, and dentate gyrus (DG)) were assessed on sections taken 17 mm anterior to stereotaxic zero. Neuronal (NeuN), oligodendroglial (Olig-2, CNPase) and microglial (Iba1) cells were counted on stained sections by light microscopy at 40x magnification on a Nikon 80i microscope with NIS Elements Br 4.0 software (Nikon Instruments Inc., Melville, N.Y., U.S.A.) using five fields in the striatum (three in the caudate nucleus, two in the putamen), two fields in the white matter (one intragyral, one periventricular) and one field in each of the hippocampal divisions. Healthy neurons were counted based on morphological assessment; neurons showing a pyknotic morphology were excluded^37^. For each animal, average scores across both hemispheres from two sections were calculated for each region. Counts were made by an assessor (PD) masked to treatment group.

### Statistics

For between group comparisons of blood chemistry and histology, two-way analysis of variance (ANOVA) for repeated measures was performed using SPSS v23 (SPSS, Chicago, IL, USA). If a significant effect was found, the effect of group was further investigated using the Fisher’s protected least significant difference (LSD) post-hoc test. The correlation between number of identified sharp wave transients at different times in the first 4 hours after occlusion and numbers of surviving neurons (NeuN-positive neurons) after 7 days recovery was evaluated by linear regression using MATLAB. For the correlation coefficients, + and – signs indicate the direction of correlations. Correlation coefficients of >0.80 were considered to be strong relationships explaining more than 64% of variance. Statistical significance was accepted when (P < 0.05). Histological and biochemical data are presented as mean ± SEM. A sensitivity and selectivity analysis was used to assess the performance of the WT-Type-2-FLS classifier.

## Results

### Fetal biochemistry and hemodynamics

The groups were not different for any measurement in the baseline period (Table 1). Umbilical cord occlusion was associated with profound hypoxia, hypercapnia, mixed respiratory and metabolic acidosis and hypoglycemia. Bradycardia and hypotension developed during occlusion; at the last minute of occlusion, fetal heart rate was 56.4 vs. 189.5 bpm and mean arterial pressure 10.2 vs. 35.6 mmHg (asphyxia vs. control, P < 0.001). At post-mortem brain weight was significantly lower in the asphyxia group than the sham control group (28.5 ± 1.1 vs 33.6 ± 0.5 g). There was no significant differences in body weight between groups (1888 ± 126 vs 2039 ± 127 g).

**Table 1:** This table shows the changes in fetal pre-ductal arterial pH, blood gases, lactate and glucose values before (baseline), during (5 and 17 minutes) and after-HI (+10 minutes, 1, 2, 4, and 6 hours).

	Group	Baseline	5 min	17 min	+10 min	+1 h	+2 h	+4 h	+6 h
pH	Control	7.38 ± 0.0	7.38 ± 0.0	7.38 ± 0.0	7.38 ± 0.0	7.38 ± 0.0	7.38 ± 0.0	7.38 ± 0.0	7.38 ± 0.0
	Asphyxia	7.39 ± 0.0	7.03 ± 0.0^#^	6.84 ± 0.0^#^	7.16 ± 0.0^#^	7.30 ± 0.0^#^	7.35 ± 0.0	7.40 ± 0.0*	7.40 ± 0.0
PaCO_2_ (mmHg)	Control	47.1 ± 0.3	43.8 ± 1.1	46.4 ± 1.0	45.2 ± 1.1	44.4 ± 1.5	49.0 ± 0.6	45.5 ± 1.0	47.0 ± 1.6
	Asphyxia	48.1 ± 0.7	107.0 ± 5.0^#^	143.2 ± 1.8^#^	55.0 ± 2.1^‡^	43.0 ± 1.0	44.0 ± 1.3*	43.0 ± 1.3	48.0 ± 0.7
PaO_2_ (mmHg)	Control	24.1 ± 1.3	24.0 ± 1.3	23.1 ± 1.4	24.0 ± 1.1	24.0 ± 1.5	23.0 ± 1.4	21.3 ± 1.3	23.0 ± 0.9
	Asphyxia	23.5 ± 1.5	6.0 ± 1.0^#^	6.4 ± 1.2^#^	33.3 ± 2.1^‡^	30.3 ± 0.4*	24.3 ± 1.2	27.0 ± 2.0	26.0 ± 2.4
Lactate (mmol/L)	Control	0.9 ± 0.1	0.8 ± 0.0	0.9 ± 0.1	0.8 ± 0.1	0.9 ± 0.1	1.0 ± 0.1	0.9 ± 0.1	0.9 ± 0.1
	Asphyxia	1.0 ± 0.1	4.4 ± 0.2^#^	6.8 ± 0.5^#^	6.3 ± 0.3^#^	3.3 ± 0.4^#^	1.5 ± 0.1^#^	2.3 ± 0.4^†^	2.3 ± 0.3^‡^
Glucose (mmol/L)	Control	1.0 ± 0.1	1.0 ± 0.1	1.0 ± 0.1	1.0 ± 0.1	1.2 ± 0.1	1.2 ± 0.1	1.1 ± 0.1	1.2 ± 0.1
	Asphyxia	1.0 ± 0.1	0.3 ± 0.1^#^	0.8 ± 0.1	1.8 ± 0.2^‡^	1.4 ± 0.1	1.9 ± 0.1	1.3 ± 0.2	1.5 ± 0.1

Data are mean ± SEM, *P < 0.05, ^†^P < 0.01, ^‡^P < 0.005, ^#^P < 0.001, control vs. HI.

### Immunohistochemistry

**Figure 3:**
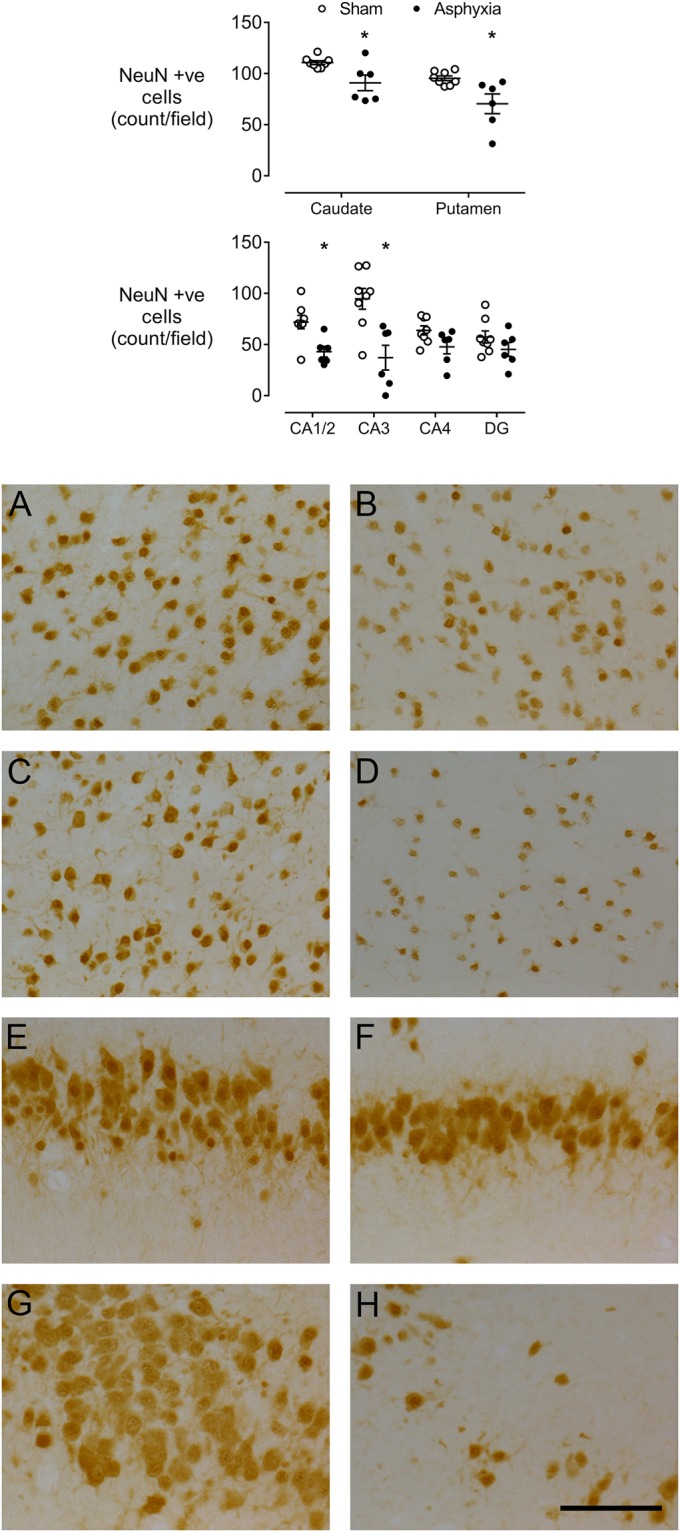
Top: vertical scatter plots of NeuN positive neuronal counts in the sham control (white circles) and asphyxia (black circles) groups in the caudate nucleus, putamen, cornu ammonis (CA) regions of the hippocampus and dentate gyrus (DG). Data are mean ± SEM. *P < 0.05. The bars show mean ± SEM. Bottom: representative photomicrographs of neuronal survival (NeuN-positive cells) in sham control (left panels) and asphyxia (right panels) groups in the caudate nucleus (**A**,**B**), putamen (**C**,**D**), CA1/2 (**E**,**F**) and CA3 (**G**,**H**). All images were taken at 40x magnification. Scale bar 20 µm.

Asphyxia was associated with significant loss of NeuN positive cells in the caudate nucleus and putamen of the striatum (P < 0.01 vs sham controls, Fig. 3), and the CA1-2 (P < 0.01) and CA3 (P < 0.005) regions of the hippocampus, but no significant change in the CA4 (P = 0.062) and the dentate gyrus (P = 0.08).

**Figure 4:**
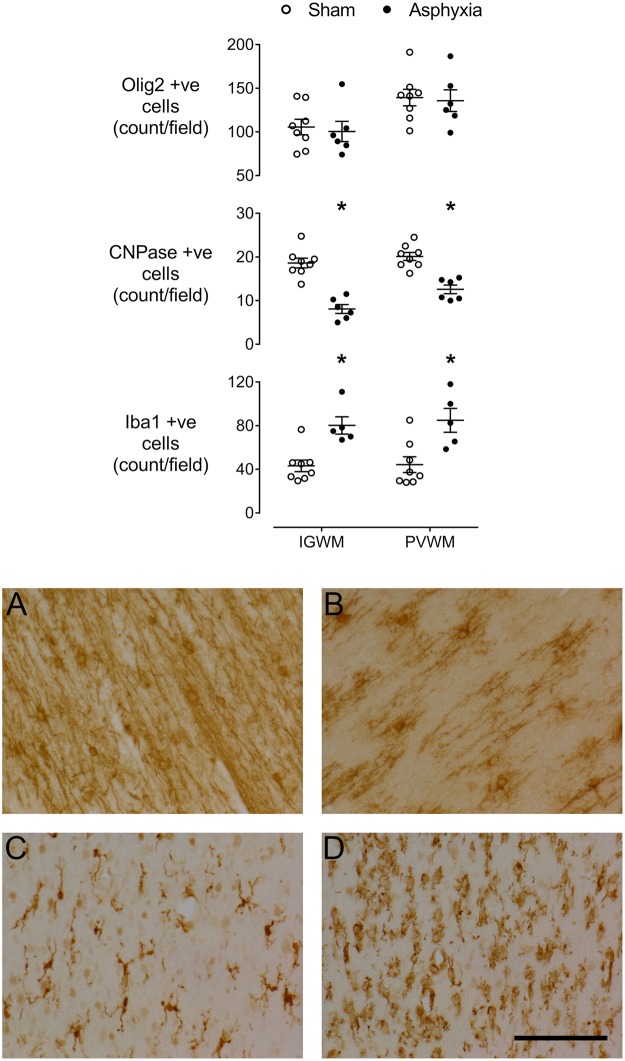
Top: vertical scatter plots showing numbers of Olig-2-positive (total) oligodendrocytes, 2′,3′-Cyclic-nucleotide 3′-phosphodiesterase (CNPase)-positive (immature/mature) oligodendrocytes and microglia (ionized calcium binding adaptor molecule 1 (Iba1)-positive) in the intragyral white matter (IGWM) and periventricular white matter (PVWM) of sham control (white circles) and asphyxia fetuses (black circles). The bars show mean ± SEM. *P < 0.05. Bottom: Representative photomicrographs of CNPase-positive immature/mature oligodendrocytes in the sham control (**A**) and asphyxia (**B**) groups, and Iba1-positive microglia in the sham control (**C**) and asphyxia (**D**) groups. All images were taken at 40x magnification from the intragyral white matter. Scale bar 20 µm.

There was no significant change in numbers of Olig-2-positive oligodendrocytes after asphyxia compared to sham controls (Olig-2 positive, Fig. 4), whereas there was a marked loss of CNPase positive cells in both the intragyral and periventricular white matter (P < 0.001). Moreover, numbers of Iba1 positive microglia were increased in both white matter regions (P < 0.005).

### Sharp wave and neuronal loss correlations

**Figure 5:**
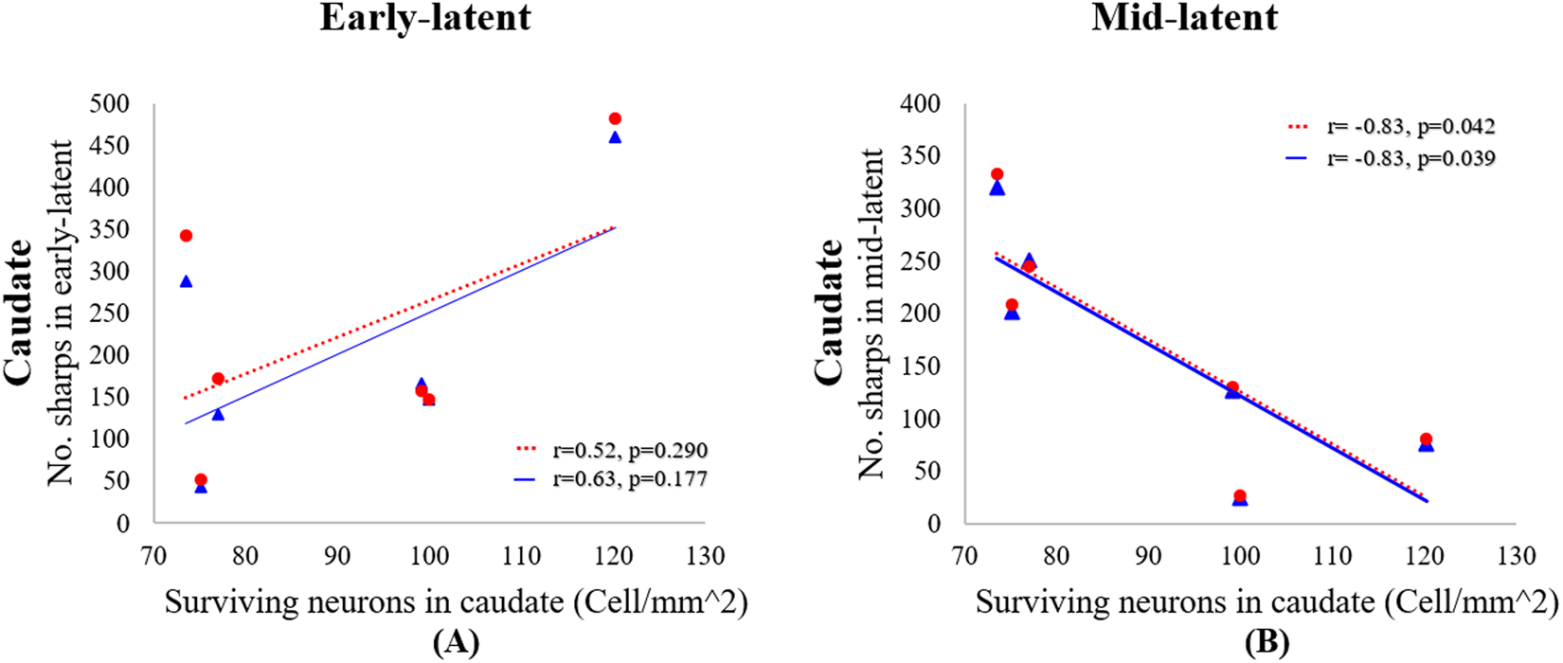
The relationship between the number of post-occlusion sharp wave epileptiform transients from (**A**) 0 to 2 hours (early-latent phase) and (**B**) 2 to 4 hours (mid-latent phase) vs the number of surviving neurons (cell/mm2) in the caudate nucleus. The solid blue lines and triangles correspond to manual counts by an expert masked to the experimental group, whereas the dotted red lines and circles represent the automatic count of the sharp waves using the WT-Type-2-Fuzzy classifier.

We initially assessed the correlation between numbers of NeuN-positive neurons in all brain regions and numbers of sharp waves as counted by both manual and automatic methods for the first two, 2 hour, epochs that represent the early and mid-latent phases respectively (Table 2). Within the brain regions showing reduced numbers of NeuN-positive neurons in the present study, significant correlations were observed only for the caudate nucleus (Table 2, Fig. 5).

**Table 2:** Correlation coefficients for numbers sharp waves in 2 hour epochs in the first 4 hours after HI vs.

	No. of Sharps – early latent				No. of Sharps – mid latent			
	Manual		Auto		Manual		Auto	
	Corr.	P	Corr.	P	Corr.	P	Corr.	P
Caudate	0.63	0.177	0.52	0.290	−0.83*	0.039*	−0.83*	0.042*
Putamen	0.58	0.226	0.51	0.296	−0.48	0.331	−0.44	0.379
CA1/2	−0.58	0.225	−0.53	0.276	0.09	0.861	0.05	0.927
CA3	0.64	0.171	0.61	0.201	−0.39	0.450	−0.35	0.496

neuronal survival in selected striatal and hippocampal brain regions 7 days after HI.

Corr.: correlation. P: P value. *P < 0.05. Manual: manually counted sharp waves. Auto: automatic classifier.

### Early-latent phase (0–2 hours)

There was no significant correlation between the numbers of sharp waves for either the manual and automated method for the 1 or 2 hour epochs and numbers of NeuN-positive neurons in the caudate nucleus. However, within the 30 minute epochs there was a strong positive correlation in the first 30 minutes after HI between the number of sharp waves and numbers of NeuN-positive neurons after 7 days for both the automated (r =+0.80, P < 0.05) and manual counts of sharp waves (r =+0.87, P < 0.05). Analysis using 10 minute epochs showed significant correlations between manual counts and numbers of NeuN-positive neurons at 30–40 minutes (r = +0.83, P < 0.05) and 40–50 minutes (r =+0.81, P < 0.05).

### Mid-latent phase (2–4 hours)

For the whole 2 hour epoch, there was a negative correlation between the number of sharp waves and numbers of NeuN-positive neurons in the caudate nucleus for both the manual (r = −0.83, P < 0.05) and automated sharp wave counts (r = −0.83, P < 0.05). For one hour epochs there was a negative correlation in the 4^th^ hour after HI for manual (r = −0.87, P < 0.05) and automated counts (r = −0.86, P < 0.05). For 30 minute epochs a correlation was seen at 3.5 hours for both manual (r = −0.82, P < 0.05) and the automated counts (r = −0.82, P < 0.05). By contrast, the 10 minutes epochs showed a significant correlation only at 200–210 minutes for both manual and automated counts (r = −0.85, P < 0.05).

## Discussion

This study supports the potential utility of automated counts of epileptiform transients as a biomarker for early prediction of subcortical neuronal loss in the preterm brain after a significant period of asphyxia induced by umbilical cord occlusion *in utero*. Greater numbers of sharp wave transients by approximately 3 to 4 hours after HI were strongly associated with reduced numbers of NeuN-positive neurons in the caudate nucleus. This finding was able to be replicated on temporal epochs as short as 10 minutes. Unexpectedly, we found that greater numbers of transients in the first 30 minutes after HI were associated with greater neuronal survival, demonstrating that the temporal evolution of transients is highly affected by the severity of HI. This highlights the importance of continuous monitoring after HI to assess the pattern of evolution of transients, rather than relying on short “snapshots” in time. Finally, this study demonstrates that by combining signal processing approaches^23,24^, it is possible to automate the accurate detection and quantification of sharp waves in the latent phase in real-time to match the precision of manual counting methods.

Similarly to previous studies from our team and others^38–42^, umbilical cord occlusion for 25 minutes was associated with significant subcortical brain injury as shown by neuronal loss in the striatum and regions of the hippocampus, and with loss of immature/mature oligodendrocytes and induction of microglia in the periventricular and intragyral white matter. There was no significant net loss of Olig-2 positive oligodendrocytes in the intragyral and periventricular white matter after 7 days recovery despite marked loss of CNPase positive oligodendrocytes. This shows that there was a shift to a greater proportion of immature oligodendroglia and is highly consistent with previous studies showing maturational arrest of oligodendrocytes in the neonatal rat after HI^43^, fetal sheep after severe asphyxia^38,39^, and in premature infants with diffuse white matter injury^44^. Asphyxia in turn was associated with initial suppression of EEG power for approximately 4 to 6 hours in a latent phase of HI injury, followed by delayed onset of seizures^39^.

Previous studies suggest that optimal recovery of cerebral metabolism after such severe HI is only present for the first few hours^6,7^. Pre-clinical studies in term-equivalent animals have shown that therapeutic hypothermia could prevent the majority of cell death when started within 3 hours after HI^45^, but hypothermic neuroprotection was largely lost if the start time of treatment was delayed until after ~6 hours^46^. Clinically, meta-analysis of large randomized controlled trials have confirmed that therapeutic hypothermia is effective if it is started as early as possible in the first 6 hours of life^9^. These trials suggest that despite significant improvement in survival without disability, nearly half of infants with HIE will still die or survive with disability after hypothermia treatment. In part this appears be due to the fact that sometimes the period of HI may begin well before birth, and thus may have evolved beyond the optimal treatment window by the time therapeutic hypothermia has been initiated^11^. This problem of injury having markedly evolved by the time of birth may be an even greater issue for preterm infants, who are at much greater risk of HI before birth^10^.

Thus, to initiate effective potential neuroprotective interventions we need biomarkers that can be used continuously to discriminate between the phases of injury, and preferably specific epochs within those phases, to allow treatment to start as early as possible. EEG monitoring of epileptiform transients offers the potential for a continuous, non-invasive biomarker of evolving neuronal loss^47^. Cot-side aEEG recordings have already shown that patterns such as prolonged EEG suppression and burst suppression patterns are associated with adverse neural outcomes^18,47^. However, at present EEG monitoring in preterm infants is often started days after birth, and the ‘early’ predictive capacity of EEG recordings usually refers to 24–72 hours^18^. Animal studies show that EEG amplitude is initially suppressed in the latent phase as part of endogenous neuroprotective inhibition, regardless of the severity of brain injury^28,48^. Thus, it is not surprising that EEG suppression early in the latent phase after HI does not correlate well with neural outcomes. Where a correlation has been found, it has usually been towards the end of the latent phase^12,49^.

By contrast, we have previously reported that changes in EEG frequency may better predict outcomes at an earlier stage and, critically, that the development of epileptiform transients (including spikes, sharp waves, and slow waves) underpin these changes in EEG frequency^6^. We have previously demonstrated that epileptiform transients only occur in preterm fetuses that go on to develop brain injury^28^, and that total numbers of all transients (spikes, sharp waves, and slow waves) during the latent phase correlated with striatal neuronal loss^29^. These data are consistent with clinical observations that the presence of many epileptiform transients is associated with adverse neurological outcomes in newborns^26,50^. However, these studies did not evaluate the temporal evolution of transients.

The current study evaluated one specific type of epileptiform transient, the sharp wave, because clinically this waveform is frequently assessed in formal EEG monitoring^51^. Clinically, increased numbers of sharp waves, and the appearance of poly-waves or runs of transients, are associated with brain lesions and adverse neurodevelopmental outcomes in term and preterm infants^27,52^. We have previously established that WT-Type-2-FLS is effective for automatic identification of sharp wave transients^24^, and in this study we have combined this technique with a stereotypic evolving micro-scale seizure filter to reduce false detection of sharp waves^23^.

The present study confirms a similar pattern of subcortical neuronal loss, including the striatum and hippocampus, to that seen in preterm infants after severe asphyxia^53^. Subsequent impaired development of these grey matter regions is associated with impaired motor and cognitive skills, and increased risk of behavioral abnormalities in later life^54–56^. Our data demonstrate that sharp waves in the middle of the latent phase after HI correlated with neuronal loss in the caudate nucleus of the striatum, but not other subcortical regions. This likely reflects the fact that evolution of injury is not uniform, but evolves at different rates between regions^57^. Caudate injury in part relates to vulnerability to expression of neuronal nitric oxide synthase likely produced by adjacent cells, which is associated with oxygen free radical production, glutamate receptor activity and calcium entry into the cells^58^. The caudate nucleus can be selectively protected by nitric oxide inhibition during HI^59^, with evidence that this helps stabilize cell function and reduce seizures^31^. However, we must also consider that other types of epileptiform transients may better predict injury in the hippocampus and putamen^6^. Further work is required to resolve this question.

Intriguingly, we observed a *positive correlation* between sharp wave counts and neuronal survival in the caudate nucleus in the first 30 minutes after HI. This finding suggests, for the first time, that the pattern of evolution of epileptiform transient activity may be highly affected by the severity of neuronal loss, such that fetuses with less neuronal loss showed an earlier, but transient, increase in epileptiform activity, compared with fetuses with more severe neuronal loss. Speculatively, this transient increase that was seen only during the first 30 minutes after HI may have been mediated by earlier restoration of function of less severely affected neurons in the caudate nucleus. It must be remembered that not all post-ischemic EEG activity should be interpreted as pathogenic. Alternatively, it may reflect reduced potential for spreading depolarization in the presence of fewer irreversibly injured neurons. It will be important to confirm in future studies whether similar patterns of injury are seen after different types and patterns of HI^60^.

In the mid-latent phase, 2 to 4 hours after HI in the present study, there was a strong negative correlation between sharp waves and numbers of surviving neurons 7 days after HI in the caudate nucleus. This finding suggests that injured neurons that were destined to die were able to generate sharp waves in this phase. By contrast, we speculate that neurons that initially recovered had become quiescent as part of endogenous neuroprotection, which in part acts by suppressing neuronal activity to reduce metabolic demand^61^. It is during mid-latent phase that the greatest numbers of transients occurred, and that levels of mitochondrial cytochrome oxidase begins to fall^6^. Moreover, we have previously demonstrated that increasing epileptiform transient numbers is associated with increased injury^19^ and suppressing transients with decreased injury^29,62^. Thus, increased numbers of sharp waves not only signal cellular dysfunction, but potentially may contribute to evolving injury by metabolically stressing injured cells.

In summary, the present data demonstrate the utility of EEG sharp waves in predicting neural injury, and confirm that increasing numbers of sharp waves, superimposed on a suppressed EEG background, and in the absence of high amplitude seizures, marks the early to mid-latent phase. We have shown that these sharp waves are driven at least in part by evolving cell injury in the caudate nucleus and was associated with reduced total numbers of NeuN-positive neurons after 7 days recovery. Further studies are required to determine the utility of sharp waves in predicting injury to other important subcortical regions such as the thalamus and to assess whether the effect of interventions such as therapeutic hypothermia on these sharp waves can provide information on response to treatment^29^. We have also demonstrated that very early sharp waves in the first 30 minutes after HI are not necessarily pathogenic, but speculatively may reflect restoration of cellular homeostasis in more mildly injured cells. This marked impact of severity of neuronal loss on the pattern of evolution of sharp wave transients after HI highlights the need for early and continuous EEG monitoring to enable the pattern of transient activity to be reliably documented. Finally, our study demonstrates that the potential to automate measurement of sharp waves in windows as short as 10 to 30 minutes.
